# Gut microbiota and metabolomic changes across preterm stages: potential associations with bronchopulmonary dysplasia

**DOI:** 10.1128/spectrum.02740-25

**Published:** 2026-02-06

**Authors:** Chunfang Gu, Mingzhao Han, Xiuling Chen, Yuting Liu, Guozhen Jian, Qiongyu Qin, Huaiyuan Yin, Lixia Zhou, Dong Cai, Li Zhang, Danhong Wang, Peng Li

**Affiliations:** 1 Department of Pediatrics, Haikou Affiliated Hospital of Central South University Xiangya School of Medicine, Haikou, China; 2 Ministry of Education Key Laboratory for Ecology of Tropical Islands, College of Life Sciences, Hainan Normal University 12389, Haikou, China; 3 Key Laboratory of Tropical Animal and Plant Ecology of Hainan Province, College of Life Sciences, Hainan Normal University 117783, Haikou, China; 4 Department of Neonatology, Hainan General Hospital 26496, Haikou, Hainan, China; 5 Department of Neonatology, Hainan Women and Children Hospital, Haikou, China; LSU Health Shreveport, Shreveport, Louisiana, USA

**Keywords:** biomarkers, dysbiosis, gut-lung axis, microbial succession, multiomics

## Abstract

The coordinated post-natal development of the gut microbiome and metabolome is essential for preterm infant health, yet its disruption is increasingly linked to adverse outcomes such as bronchopulmonary dysplasia (BPD). In this study, we performed an integrated multiomics analysis of fecal samples collected from preterm infants to characterize temporal changes in gut microbial and metabolic profiles and explore their potential associations with BPD development. This study observed a distinct trajectory of the phylum Bacteroidota as a hallmark of normal gut maturation, with its abundance progressively declining across non-BPD infants. In contrast, infants who later developed BPD exhibited early depletion followed by irregular enrichment of Bacteroidota. Correlation analysis revealed that *Streptococcus* abundance was positively associated with elevated cysteic acid, a metabolite linked to oxidative stress. Together, these findings suggest that altered Bacteroidota succession and *Streptococcus*-associated oxidative imbalance may reflect early microbial-metabolic perturbations in infants at risk of BPD. This work provides preliminary, hypothesis-generating insights into gut-associated signatures potentially relevant to BPD pathogenesis.

**IMPORTANCE:**

Bronchopulmonary dysplasia (BPD) remains a leading cause of morbidity in preterm infants, yet early biomarkers and targeted preventive strategies are limited. By integrating microbiome and metabolome data from a pilot cohort, this study identified patterns of disrupted Bacteroidota succession and *Streptococcus*-associated oxidative stress that are associated with BPD risk. These findings highlight the gut as a potential extrapulmonary contributor to disease susceptibility and support early risk assessment and guide future microbiome-targeted interventions in preterm infants.

## INTRODUCTION

Preterm birth, defined as delivery before 37 weeks of gestation, affects approximately 15 million newborns annually, with a global incidence of around 11% (1, 2). The associated high rate of mortality and morbidity makes it a major global health concern (3). Notably, both the gut and lungs originate from the primitive foregut during embryonic development (4). Recent advances in microbiome research have led to the proposal of a “gut-lung axis,” a bidirectional communication network between the gastrointestinal and respiratory systems (5, 6). The gut-lung axis is thought to mediate interactions via microbial components, bacterial metabolites, and immune cell trafficking (7). These signals can travel through circulatory routes, neural pathways, or even direct microbial translocation. As a key component of this axis, the gut microbiota, often regarded as a “hidden organ,” plays essential roles in host development, immune regulation, and disease pathogenesis (8, 9). Increasing evidence suggests that gut microbial composition can influence pulmonary immune responses (10), underscoring the importance of characterizing the gut microbiome in respiratory diseases such as inflammation and infection (11). Furthermore, the establishment of the gut microbiome in early life is a critical window influencing these clinical outcomes. Unlike full-term infants, the successional trajectory of the preterm gut microbiome is often characterized by “arrested development” or “dysbiosis,” yet the underlying ecological mechanisms and functional consequences of this deviation remain poorly understood.

Bronchopulmonary dysplasia (BPD), a chronic lung disease predominantly affecting preterm infants, has been increasingly linked to early-life alterations in gut microbiota (12). Recent studies have established a significant association between neonatal BPD and gut microbiota, with BPD patients exhibiting a higher likelihood of early gut dysbiosis (13). Specifically, research has revealed diminished intestinal microbiota diversity in preterm infants with BPD compared to those without BPD by day 28 (14). Moreover, vaginally born infants with BPD demonstrated decreased levels of *Klebsiella* and *Shigella* flora in their intestines relative to non-BPD infants, and BPD infants exhibited reductions in operational taxonomic units, relative abundance, and the Shannon index of intestinal microbiota by day 28 post-birth (15). These findings suggest that disrupted microbial colonization and decreased diversity may contribute to BPD development in preterm neonates. Despite advances in neonatal care, BPD incidence continues to rise alongside increasing preterm birth rates, posing long-term health challenges (16). Probiotics such as *Bifidobacterium* have been shown to reduce the risk of necrotizing enterocolitis (NEC) and may also benefit BPD outcomes, while *Lactobacillus* has been implicated in modulating alveolar growth, but its clinical efficacy remains uncertain. The precise mechanisms by which gut dysbiosis contributes to BPD via the gut-lung axis remain poorly understood.

Previous studies have explored the relationship between gut dysbiosis and the development of BPD in preterm infants, suggesting that alterations in early microbial colonization may influence lung development through immune and metabolic pathways (17–19). These investigations provided valuable mechanistic insights, such as the role of microbial metabolites and host immune modulation in shaping pulmonary outcomes. However, most prior work relies on small cross-sectional cohorts or focuses exclusively on microbial composition without integrating metabolic profiles. In contrast, our study combines 16S rRNA-based microbiome profiling with fecal metabolomic analysis to explore the co-development of gut microbiota and metabolic function in the early post-natal period, aiming to identify microbial-metabolic signatures associated with BPD risk. This integrative approach provides a more comprehensive view of gut ecosystem maturation in preterm infants and its potential link to BPD susceptibility.

## MATERIALS AND METHODS

### Study subjects and sample collection

A total of 62 fecal samples were collected from preterm infants hospitalized in the Department of Pediatrics at Haikou People’s Hospital and the Department of Neonatology at Haikou Maternal and Child Health Hospital and Hainan Provincial People’s Hospital (Haikou, Hainan Province, China), respectively. Infants were classified into three groups based on gestational age at birth: early preterm (S1, 28–31 weeks and 6 days, *n* = 13), middle preterm (S2, 32–33 weeks and 6 days, *n* = 14), and late preterm (S3, 34–36 weeks and 6 days, *n* = 35). All fecal samples, except for a subset used in longitudinal analysis, were collected within 24 hours after birth and immediately stored at −80°C following standard protocols.

Among the total samples, six were obtained from infants later diagnosed with BPD according to 2019 criteria established by Eunice Kennedy Shriver National Institute of Child Health and Human Development Neonatal Research Network, including three samples (two of grade I and one of grade II; Table S1) collected within 24 hours of birth (BPD1) and three collected on day 28 (BPD2). To serve as time-matched controls, an additional six fecal samples were selected from non-BPD (NBPD) infants, including three randomly collected within 24 hours (NBPD1) and three on day 28 (NBPD2). These six samples originated from three non-BPD infants who were sampled at both time points. Except for samples from BPD2 and NBPD2, the remaining 56 samples (62 total minus 6 BPD samples) were from non-BPD infants, all of which were collected within 24 hours of birth. Notably, due to sampling constraints at the time of study design, most non-BPD infants were not sampled at day 28, limiting the number of longitudinal control samples to three. This sample structure allowed us to assess gut microbial differences across preterm stages and to preliminarily explore early post-natal microbial succession in BPD versus non-BPD infants. Clinical metadata, including gestational age, birth weight, delivery mode, and antibiotic exposure, were recorded and used to confirm that there were no significant differences between BPD and non-BPD groups (Tables S2 and S3). However, due to limited sample size, these variables were not included as covariates in the statistical models.

### DNA extraction and sequencing analysis

Genomic DNA was extracted from fecal samples using the QIAamp Fast DNA Stool Mini Kit (Qiagen, Hilden, Germany) following the manufacturer’s instructions. The quality and concentration of extracted DNA were assessed using a NanoDrop spectrophotometer (Thermo Fisher Scientific, USA) and confirmed via 1% agarose gel electrophoresis. Qualified DNA samples were then sent to Shanghai Applied Protein Technology Co., Ltd. (Shanghai, China) for library construction and sequencing.

The V3–V4 hypervariable regions of the bacterial 16S rRNA gene were amplified using region-specific primers (338F: 5′-ACTCCTACGGGAGGCAGCAG-3′ and 806R: 5′-GGACTACHVGGGTWTCTAAT-3′) and sequenced on the Illumina NovaSeq 6000 platform, generating 250 bp paired-end reads. Raw sequencing data were subjected to quality control (QC) and denoising using QIIME2 (v.2022.2) with default parameters, including trimming of low-quality bases, merging of paired-end reads, and removal of chimeric sequences. Amplicon sequence variants (ASVs) were identified using the DADA2 plugin within QIIME2. To reduce background noise, ASVs with a total abundance of fewer than 10 reads across all samples were removed prior to downstream analysis.

### Metabolite extraction and LC-MS/MS analysis

Fecal metabolites were extracted following a standard protocol. Briefly, samples were slowly thawed at 4℃, and an appropriate amount of each sample was mixed with prechilled methanol/acetonitrile/water (2:2:1, vol/vol/vol). The mixture was vortexed, sonicated at low temperature for 30 min, and then incubated at −20℃ for 10 min. After centrifugation at 14,000 × *g* for 20 min at 4℃, the supernatant was collected and vacuum dried. For LC-MS analysis, the dried extract was reconstituted in 100 μL of acetonitrile/water (1:1, vol/vol), vortexed, and centrifuged again at 14,000 × *g* for 15 min at 4°C. The final supernatant was used for injection.

Chromatographic separation was performed using an Agilent 1290 Infinity LC system equipped with a hydrophilic interaction chromatography column (25℃ column temperature, flow rate: 0.5 mL/min, injection volume: 2 μL). The mobile phases consisted of 25 mM ammonium acetate and 25 mM ammonia hydroxide in water (A) and acetonitrile (B). The gradient elution was programmed as follows: 0–0.5 min, 95% B; 0.5–7.0 min, 95%–65% B; 7–8 min, 65%–40% B; 8–9 min, hold at 40% B; 9.0–9.1 min, 40%–95% B; and 9.1–12.0 min, hold at 95% B. Samples were kept at 4℃ in the autosampler throughout the analysis. Mass spectrometry was performed on an AB SCIEX TripleTOF 6600 system using electrospray ionization in both positive and negative ion modes. The MS acquisition included both full-scan (MS1) and data-dependent MS/MS (MS2) analyses. To ensure data quality and instrument stability, samples were analyzed in a randomized order, and pooled QC samples were injected periodically throughout the batch.

### Statistical and bioinformatic analyses

All statistical analyses and visualizations were performed using R software (v.4.5.1) unless otherwise specified. For microbiome data, alpha diversity was calculated using the Shannon index, and differences in diversity metrics among groups were assessed using Kruskal-Wallis tests (S1, S2, and S3) or Wilcoxon rank-sum tests (BPD-NBPD). Beta diversity was calculated based on Bray-Curtis dissimilarity and visualized using non-metric multidimensional scaling (NMDS) ordination implemented in the *vegan* package. Statistical significance of community structure differences among groups was assessed by permutational multivariate analysis of variance. Venn diagrams were generated to visualize the number of shared and unique ASVs across groups. Taxonomic composition at the phylum, class, and order levels was visualized using stacked bar plots. Linear discriminant analysis effect size (LEfSe) was used to identify significantly enriched taxa among groups using a threshold LDA score of >2.0 and raw *P* value of <0.05. Co-occurrence networks were analyzed using Spearman correlation coefficients based on the top 50 most abundant genera, with a correlation threshold of |*r*| = 0.4. Co-occurrence network and topological parameters (average degree, diameter, clustering coefficient, and modularity) were computed and visualized in Gephi (v.0.9.7). Functional prediction of microbial communities was performed based on 16S rRNA gene data using PICRUSt2 and annotated against the KEGG database. For untargeted metabolomics data, principal component analysis (PCA) was used to visualize metabolic variation among groups. Differential metabolites were identified using volcano plots based on fold change and statistical significance (*P* < 0.05). KEGG pathway enrichment analysis was performed to explore the functional implications of significantly altered metabolites. For metabolomics analysis, raw LC-MS data were first converted to the .mzXML format using ProteoWizard (v.3.0). Peak alignment, retention time correction, and peak area extraction were performed with the XCMS package (v.3.12.0). The resulting data matrix was then subjected to metabolite structure annotation based on accurate mass and MS/MS spectra against in-house and public databases (HMDB and METLIN). Putative identification levels were assigned following the Metabolomics Standards Initiative framework. Data preprocessing included removal of ion features with >50% missing values, *K*-nearest neighbor imputation for remaining missing values, and filtering of features with relative standard deviation of >50% in QC samples. Data were log_2_-transformed and pareto-scaled prior to statistical analysis. Differential metabolites between groups were determined by univariate analysis (Wilcoxon rank-sum test) with Benjamini-Hochberg FDR correction, using thresholds of |fold change| >1.5 and FDR-adjusted *P* < 0.05. Quality control evaluation included six standard QC criteria (e.g., retention time stability, mass accuracy, peak shape, and reproducibility across QC samples). To explore potential interactions between the gut microbiota (genus level) and metabolites, Spearman correlation analysis was performed between the relative abundance of microbial genera and the intensities of differential metabolites using heatmaps.

## RESULTS

### Analysis of microbial community characterization

Venn diagram analysis revealed distinct ASV distribution patterns across gestational stages and clinical conditions (Fig. S1; File S1). While early (S1) and middle (S2) preterm infants showed 313 and 445 unique ASVs, respectively, the late preterm group (S3) displayed a markedly higher number (828), reflecting increased microbial diversification with gestational maturity (Fig. S1a). A similar trend was observed in relation to disease status: BPD1 samples contained 98 unique ASVs, whereas NBPD1 harbored 159 (Fig. S1b), underscoring the potential influence of clinical condition on gut microbial composition. Temporal comparisons further revealed divergent microbial dynamics, with NBPD infants showing more pronounced shifts between time points than their BPD counterparts (Fig. S1c and d).

### Gut microbial community abundance

**Fig 1:**
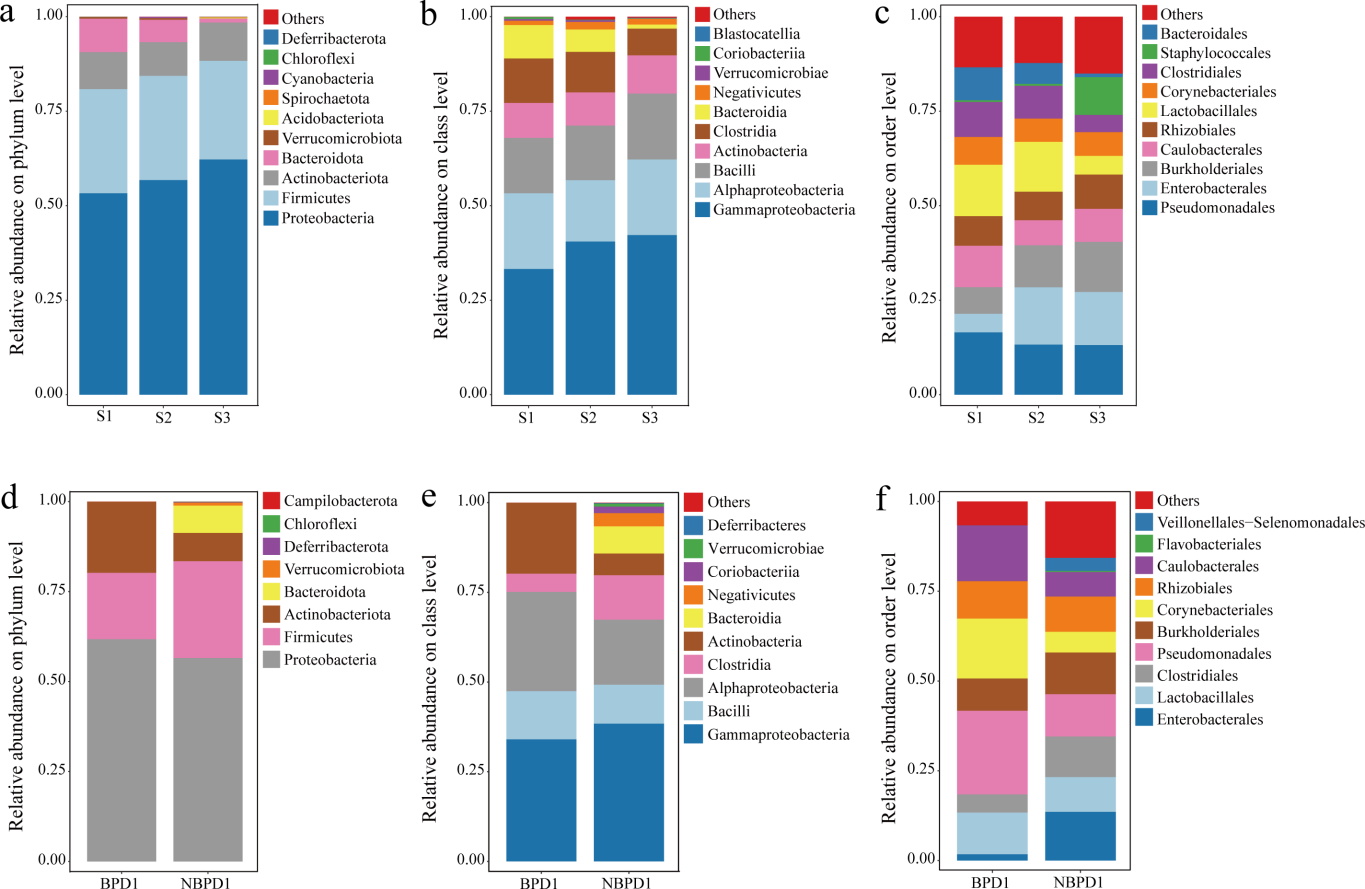
Composition of gut bacterial communities at different taxonomic levels across preterm stages and disease status. Relative abundances of bacterial taxa at the phylum (**a**), class (**b**), and order (**c**) levels among early (S1, *n* = 13), middle (S2, *n* = 14), and late (S3, *n* = 35) preterm infants. Relative abundances of bacterial taxa at the phylum (**d**), class (**e**), and order (**f**) levels in infants with BPD1 (*n* = 3) and NBPD1 (*n* = 3). Only the top 10 most abundant taxa at each taxonomic level are shown. Taxa with relative abundances below the threshold are grouped as “others.”

Analysis of gut microbial abundance across different taxonomic levels revealed stage-specific and disease-associated compositional shifts (Fig. 1). At the phylum level, Proteobacteria, Firmicutes, and Actinobacteriota dominated the microbiota across all three gestational stages (S1, S2, and S3), accounting for over 75% of the community (Fig. 1a). Among these, Proteobacteria showed a notable increase with advancing gestational age, suggesting its key role during early gut development. In contrast, Bacteroidota exhibited a marked decline from 8.86% in S1 to 1.07% in S3. A more dramatic reduction was observed in disease states: in BPD1 samples, Bacteroidota accounted for only 0.01%, compared to 7.59% in NBPD1 (Fig. 1c), indicating a potential association with gut health maintenance. At the class level, the microbiota was dominated by Gammaproteobacteria, Alphaproteobacteria, Actinobacteria, Clostridia, and Bacilli (Fig. 1b). Gammaproteobacteria was consistently the most abundant and increased progressively across gestational stages. Conversely, Clostridia and Bacteroidia declined notably, with Clostridia decreasing from 11.75% in S1 to 7.06% in S3. In the context of disease, Alphaproteobacteria and Actinobacteria were more enriched in BPD1 compared to NBPD1, which also exhibited higher species richness (Fig. 1e), reflecting a more diverse and stable microbial community in non-diseased infants. At the order level, Staphylococcales sharply increased in S3 (S1: 0.44%, S2: 0.51%, and S3: 9.99%), while Enterobacterales and Burkholderiales showed gradual increases with gestational maturity (Fig. 1c). In contrast, orders such as Lactobacillales, Clostridiales, and Bacteroidales decreased across stages. Comparisons between disease groups revealed that Pseudomonadales, Corynebacteriales, and Caulobacterales were more abundant in BPD1, while Veillonellales-Selenomonadales and unclassified “others” were elevated in NBPD1 (Fig. 1f).

To further investigate temporal shifts in gut microbiota among BPD infants, a second fecal sample was collected on post-natal day 28. The results showed a striking increase in Bacteroidota abundance, rising from 0.01% (BPD1) to 23.29% (BPD2), accompanied by a concurrent elevation in Firmicutes (Fig. S1a and c). In contrast, Bacteroidota abundance in NBPD1 was already 7.59% at birth (Fig. S2b and d), suggesting earlier colonization in NBPD infants. Thus, we propose that delayed establishment of Bacteroidota may serve as an early marker of microbial dysbiosis in infants at risk of BPD.

### Microbial community diversity

**Fig 2:**
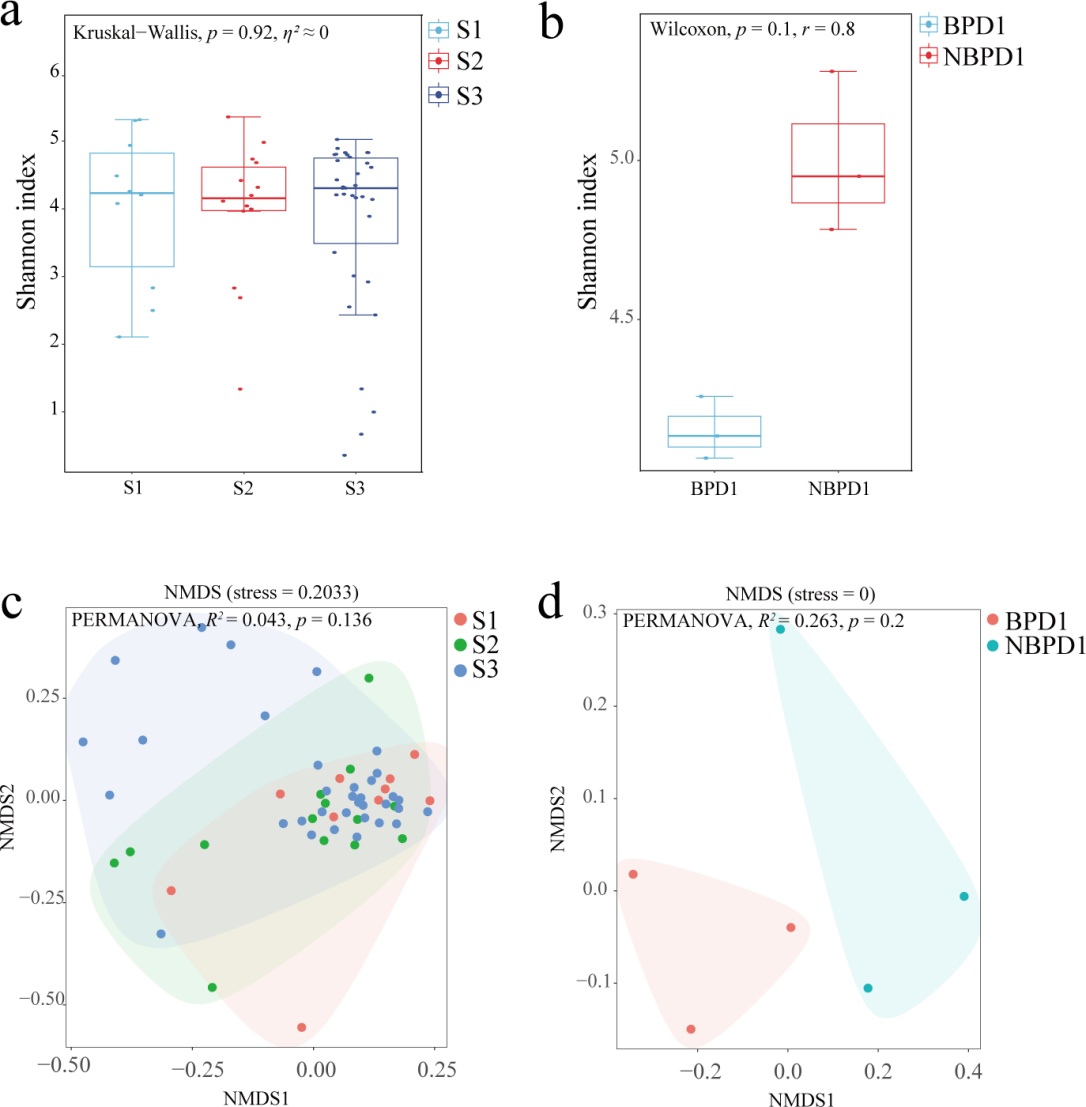
Alpha and beta diversity of gut microbiota across preterm stages and disease status. Shannon indexes of gut bacterial communities across early (S1, *n* = 13), middle (S2, *n* = 14), and late (S3, *n* = 35) preterm groups (**a**) and between BPD1 (*n* = 3) and NBPD1 (*n* = 3) (**b**). Non-metric multidimensional scaling (NMDS) based on Bray-Curtis distances illustrating differences in beta diversity among the three preterm stages (**c**) and between BPD1 and NBPD1 (**d**). Permutational multivariate analysis of variance (PERMANOVA) *R^2^* values are reported with 95% CIs estimated from permutation variance.

Alpha-diversity analysis, assessed by the Shannon index, revealed no significant differences across gestational stages (Fig. 2a) or between BPD and NBPD groups (Fig. 2b). Similarly, beta-diversity analysis based on NMDS did not demonstrate statistically significant clustering by gestational age (Fig. 2c) or disease status (Fig. 2d), although slight shifts in microbial community composition were visually apparent. These results indicate that overall microbial diversity remains relatively stable during early post-natal development and is not markedly altered by BPD. Nevertheless, the subtle compositional changes observed point toward the importance of specific taxon-level alterations rather than broad diversity shifts in the pathogenesis of BPD.

### Analysis of LEfSe and co-occurrence network

LEfSe analysis revealed significant differences in gut microbial composition across developmental stages (S1–S3) and between disease states (BPD1 versus NBPD1) (Fig. S3; File S2). Stage-specific taxa were observed at each gestational phase. In the disease comparison, several taxa were differentially enriched in BPD1 versus NBPD1, suggesting a dysbiotic signature potential associated with disease progression.

**Fig 3:**
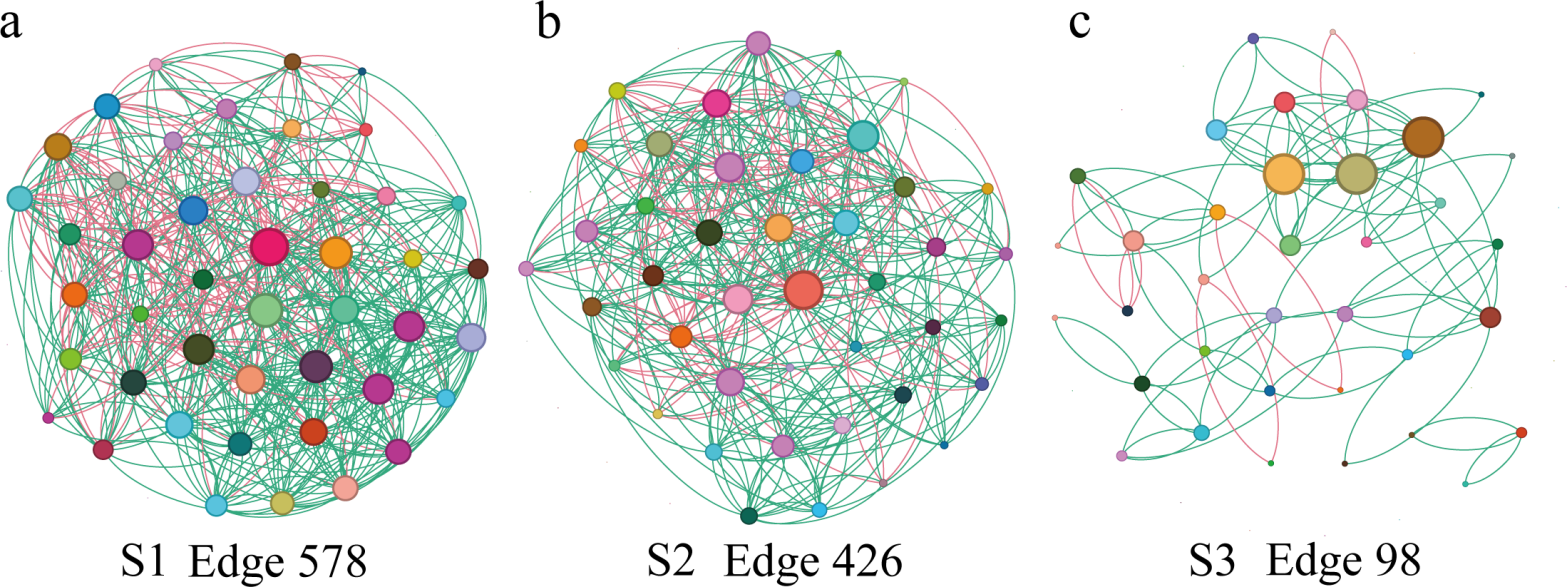
Co-occurrence networks of the top 50 bacterial genera in the gut microbiota of preterm infants at early (**a**, *n* = 13), middle (**b**, *n* = 14), and late (**c**, *n* = 35) stages. Nodes represent bacterial genera, and edges indicate significant correlations between genera. Green edges represent positive correlations, while red edges indicate negative correlations among microbial taxa, with a correlation threshold of |*r*| = 0.4.

Genus-level co-occurrence network analysis (top 50 taxa) further illustrated dynamic changes in microbial interactions (Fig. 3). In the early (S1) and middle (S2) stages, networks were densely connected, indicating complex and cooperative microbial communities (Fig. 3a and b). Key genera such as *Acinetobacter*, *Brevundimonas*, and *Pseudomonas* consistently occupied central nodes across stages, highlighting core ecological roles in the preterm gut. In contrast, the S3 network displayed reduced connectivity and low complexity (Fig. 3c), which may reflect microbial streamlining or ecosystem stabilization during maturation. As shown in Table 1, network connectivity gradually decreased from S1 to S3, as evidenced by reductions in density (from 0.292 to 0.078) and average degree (from 12.8 to 2.7). In contrast, relative modularity increased (0.278–0.573), suggesting that the initially dense but loosely structured early network progressively reorganized into more distinct and stable modules. The clustering coefficient also declined over time, indicating that microbial associations became less redundant and more specialized as the community matured. These topological changes collectively reflect ecological stabilization of the gut microbiota during post-natal development.

**TABLE 1:** Topological properties of gut microbial co-occurrence networks^*a*^

Treatment	Diameter	Density	Average degree	Relative modularity	Clustering coefficient
S1	3	0.292	12.844	0.278	0.452
S2	4	0.236	9.907	0.304	0.461
S3	12	0.078	2.722	0.573	0.261

^*a*^ Density and average degree reflect overall network connectivity; modularity and clustering coefficient indicate community segregation and internal cohesiveness.

### Metabolomic shifts across gestational stages and BPD status

**Fig 4:**
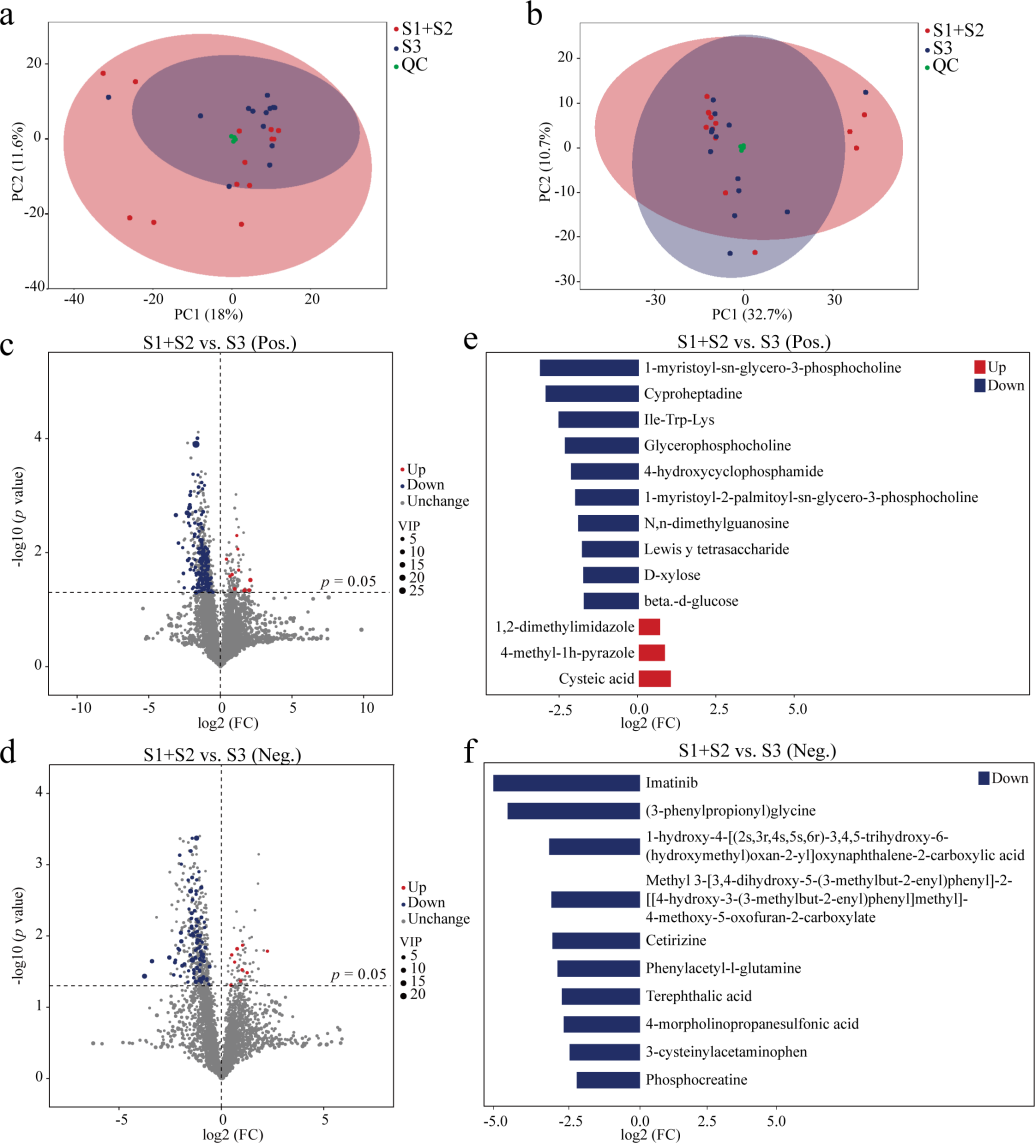
Multivariate and differential analyses of gut metabolomes in preterm infants across three developmental stages. Principal component analysis of positive (**a**) and negative (**b**) ion modes for overall metabolic profile separation. Volcano plots showing significantly regulated metabolites in positive (**c**) and negative (**d**) ion modes. Representative differential metabolites shown in butterfly plots for positive (**e**) and negative (**f**) ion modes. (S1 + S2, *n* = 27; S3, *n* = 35.) Differential metabolites between groups were determined by univariate analysis (Wilcoxon rank-sum test) with Benjamini-Hochberg FDR correction using thresholds of |fold change| >1.5 and FDR-adjusted *P* < 0.05.

To enhance statistical robustness, the S1 and S2 groups were merged due to the limited sample size in S2, allowing for a more comprehensive comparison between early-to-middle preterm (S1 + S2) and late preterm (S3) infants. Untargeted metabolomic profiling revealed distinct metabolic signatures between these groups in both positive and negative ionization modes (Fig. 4; File S3). PCA demonstrated clear separation between S1 + S2 and S3 in both modes (Fig. 4a and b), indicating substantial shifts in global metabolic landscapes with advancing gestational age. Volcano plot analysis identified 175 significantly altered metabolites in the positive ion mode, including 10 upregulated and 165 downregulated in S3 compared to S1 + S2 (Fig. 4c; File S4). In the negative mode, 103 metabolites were differentially expressed, with 9 upregulated and 94 downregulated (Fig. 4d; File S5). Butterfly plots illustrated the magnitude and direction of changes, highlighting representative metabolites. In the positive mode, several key metabolites, including IIE-Trp-Lys, 1-myristoyl-sn-glycero-3-phosphocholine, N, n-dimethylguanosine, Lewisᵞ tetrasaccharide, D-xylose, and beta-d-glucose, were markedly reduced in S3, while cysteic acid was elevated (Fig. 4e). In the negative mode, notable alterations were observed in (3-phenylpropionyl) glycine and phenylacetyl-L-glutamine (Fig. 4f).

Further metabolomic comparisons between BPD and NBPD infants were conducted in the positive ion mode (Fig. S4), as the negative mode yielded no significant differences. PCA revealed clear clustering between groups, indicating distinct metabolic profiles (Fig. S4a). Volcano plot analysis identified 29 differential metabolites, with 8 upregulated and 21 downregulated in BPD (Fig. S4b). Notably, 5-methylcytosine was elevated, while Thr-Pro and chrysomycin A were significantly downregulated in BPD (Fig. S4c). These alterations may reflect disease-related metabolic disturbances and offer potential targets for early diagnosis or therapeutic intervention.

### KEGG-based functional profiling reveals stage-specific microbial and metabolic pathway shifts

**Fig 5:**
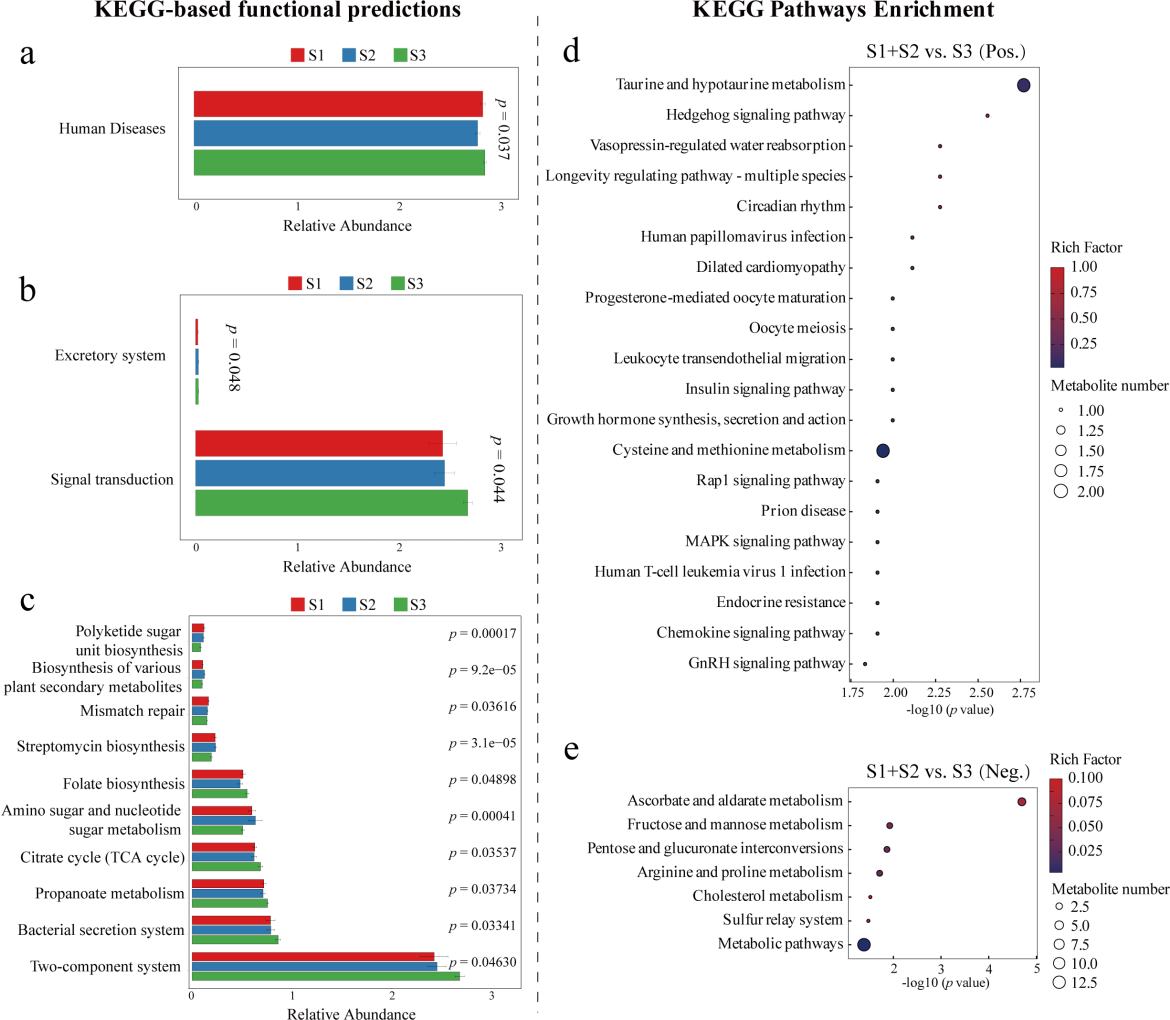
KEGG-based functional profiling of microbiome and metabolome. Predicted KEGG functional categories of the microbiome at level 1 (**a**), level 2 (**b**), and level 3 (**c**) derived from PICRUSt2 predictions. KEGG pathway enrichment of metabolites analyzed in positive (**d**) and negative (**e**) ionization modes, respectively (S1, *n* = 13; S2, *n* = 14; S3, *n* = 35).

Integrated KEGG pathway analyses based on 16S rRNA gene predictions and untargeted metabolomics revealed distinct, stage-dependent functional alterations in both microbial communities and host metabolism. At the microbiome level, predicted pathways showed significant variation across multiple KEGG hierarchy levels. At level 1 (Fig. 5a), the “human diseases” category differed significantly among gestational stages (*P* = 0.037). At level 2 (Fig. 5b), pathways related to the “excretory system” (*P* = 0.048) and “signal transduction” (*P* = 0.044) were significantly enriched. More granular changes were evident at level 3 (Fig. 5c), where functions such as “polyketide sugar unit biosynthesis,” “mismatch repair,” “streptomycin biosynthesis,” and “two-component system” were significantly enriched.

Consistent with microbial predictions, KEGG enrichment analysis of metabolomic data further supported gestational stage-specific biochemical transitions. In the positive ion mode (Fig. 5d), key pathways such as “taurine and hypotaurine metabolism” and “cysteine and methionine metabolism” were significantly enriched, indicating potential developmental regulation of sulfur-related metabolic functions. In the negative ion mode (Fig. 5e), enriched pathways were primarily related to carbohydrate and redox metabolism, including “ascorbate and aldarate metabolism,” “fructose and mannose metabolism,” and broader “metabolic pathways.”

### Correlation between key gut microbiota and fecal metabolites

**Fig 6:**
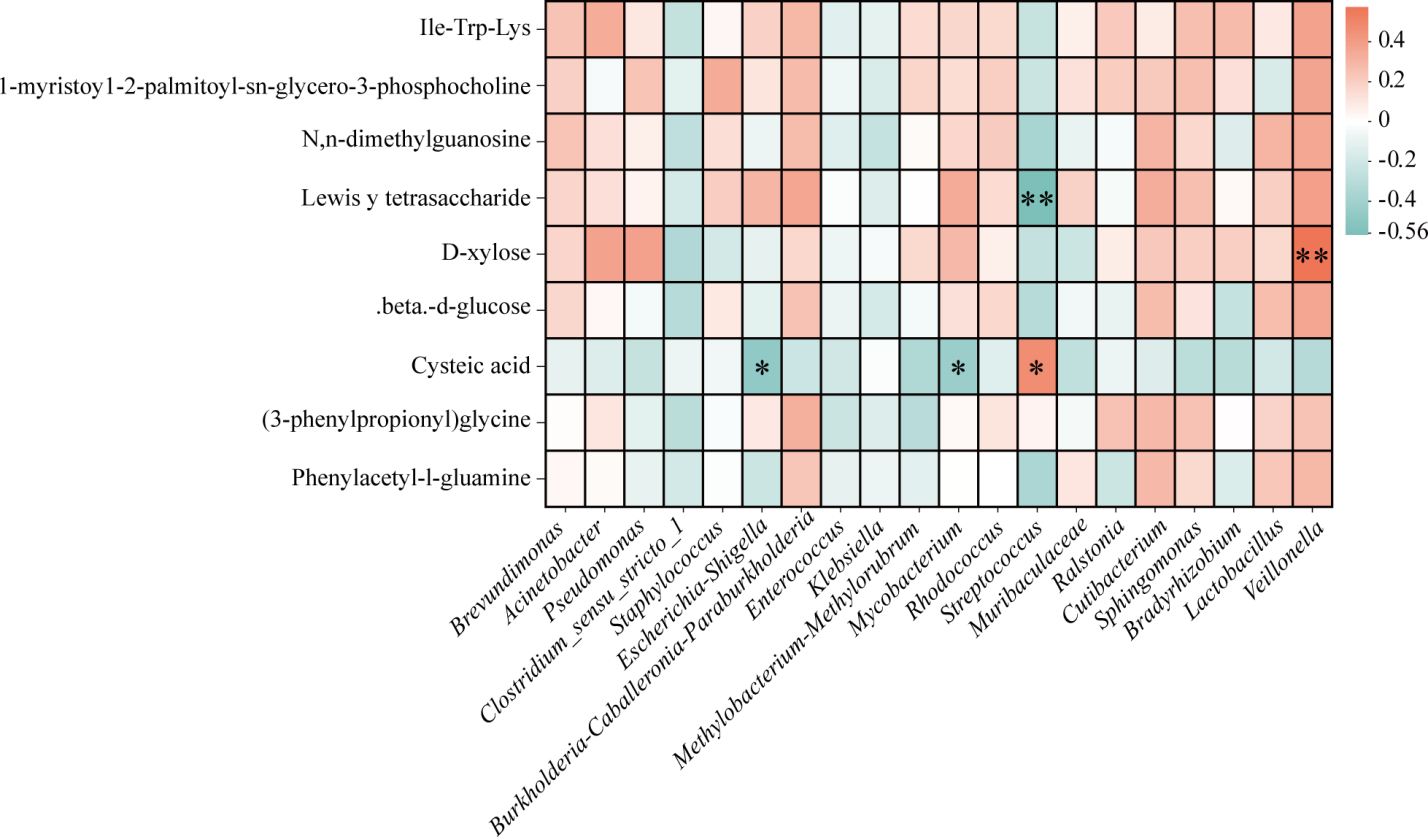
Correlation heatmaps between the top 20 bacterial genera and significantly different metabolites detected in positive and negative ion modes. *P* < 0.05 and *P* < 0.01 indicated by * and **, respectively (*n* = 62).

To explore potential functional interactions between the gut microbiota and host metabolism in preterm infants, Spearman correlation analysis was performed between the relative abundances of the top 20 bacterial genera and the levels of significantly altered fecal metabolites (Fig. 6). The analysis revealed a network of both positive and negative correlations, underscoring genus-specific metabolite relationships. Notably, *Veillonella* showed a strong positive correlation with D-xylose (*P* < 0.01), suggesting a potential role in carbohydrate metabolism. Conversely, *Streptococcus* was significantly and negatively correlated with Lewisᵞ tetrasaccharide (*P* < 0.01), implicating it in altered glycan metabolism. Levels of cysteic acid were significantly associated with multiple genera, including *Streptococcus* (*P* < 0.05), *Escherichia*-*Shigella* (*P* < 0.05), and *Mycobacterium* (*P* < 0.05). Although not statistically significant, additional trends were observed. For instance, *Acinetobacter* and *Pseudomonas* tended to show positive associations with D-xylose, while *Clostridium_sensu_stricto_1* and *Streptococcus* displayed broader patterns of negative correlation with several metabolites. These findings highlight distinct microbe-metabolite associations that may reflect functional interactions within the preterm infant gut ecosystem, offering insights into how specific bacterial taxa may contribute to or be influenced by the evolving metabolic milieu.

## DISCUSSION

### Disrupted Bacteroidota succession as an early signature of BPD risk

The gut-lung axis, a bidirectional communication network mediated through circulatory and lymphatic pathways, allows intestinal microbes to shape pulmonary immunity (20). In our study, healthy preterm infants exhibited a normative successional decline of the phylum Bacteroidota (Fig. 1a), concomitant with its established role as an early colonizer capable of metabolizing human milk oligosaccharides into beneficial short-chain fatty acids (SCFAs) to strengthen the intestinal barrier and support microbiome maturation (21, 22). In contrast, infants who later developed BPD showed pronounced disruption of this trajectory, with marked early depletion during S1 and only minimal recovery thereafter (Fig. S1d). Notably, among the three infants with BPD, the single grade 2 case exhibited persistently undetectable Bacteroidota at both time points, whereas the two grade 1 cases showed measurable increases by the later sampling stage (Fig. S2c). Although limited by small sample size, this pattern suggests a potential severity-related trend: more profound early Bacteroidota depletion may be associated with more severe BPD. Early failure of Bacteroidota colonization could impair neonatal immune maturation, promoting exaggerated inflammatory responses. In the context of underdeveloped lungs, such immune imbalance may predispose infants to the chronic inflammation characteristic of BPD (23, 24). While causality cannot be inferred, these findings highlight the need to examine early Bacteroidota establishment as a potential contributor to BPD risk in larger cohorts.

### Ecological dynamics of the preterm gut: keystone taxa outweigh overall diversity

The neonatal gut is a highly dynamic, non-equilibrium ecosystem initiated by a small set of pioneer species (25). Intriguingly, our analysis detected no significant alpha- or beta-diversity differences across post-natal stages or between BPD and NBPD groups (Fig. 2). Rather than reflecting biological similarity, this likely highlights the dominance of the “founder effect” in early-life microbial assembly (26). In such systems, timely arrival of specific keystone taxa may outweigh global diversity metrics in determining developmental trajectories (27). Our findings exemplify this concept: the key distinguishing feature between groups was not community-wide diversity but early failure to establish Bacteroidota, echoing patterns observed in NEC and sepsis where disease risk corresponds to the presence or absence of specific pathobionts rather than overall diversity (28).

Co-occurrence network analysis revealed a progressive simplification of microbial interaction networks from early to late post-natal stages (Fig. 3). This shift suggests ecological maturation toward a more stable community shaped by host immune development and epithelial differentiation, which together acts as an “ecological filter” that selects for functionally compatible taxa (29).

### The *Streptococcus*-*Veillonella* axis and its link to oxidative stress

Metabolomic profiling revealed a suppressed metabolic state in early and middle-stage infants, including decreased levels of multiple host-microbe co-metabolites [phenylacetyl-L-glutamine and (3-phenylpropionyl)glycine], key energy substrates (D-xylose and β-D-glucose), and membrane-associated lipids (1-myristoyl-sn-glycero-3-phosphocholine). Conversely, cysteic acid—an oxidative stress marker—was significantly upregulated (Fig. 4e and f). Correlation analysis further identified *Veillonella* and *Streptococcus* as forming a reciprocal metabolic axis (Fig. 6).

The genus *Veillonella*, a lactate-utilizing SCFA producer (30), showed positive associations with carbohydrate and amino acid metabolites, suggesting a link to metabolic maturation, and negative correlations with cysteic acid. In contrast, *Streptococcus* abundance (S1: 6.40%, S3: 1.72%; File S6) correlated positively with cysteic acid and negatively with all metabolites indicative of gut maturity, including Lewisᵞ tetrasaccharide. Given the known pathogenic potential of certain *Streptococcus* species recognized as potential pathobionts in preterm infants (28, 31), these findings suggest that a *Streptococcus*-dominated environment may promote inflammation and metabolic dysregulation early in life (32).

### Functional signatures of an unstable and stressed early-life gut ecosystem

Microbiome-metabolome pathway integration revealed that the early preterm gut is functionally primed for competition and environmental sensing. Enrichment of the KEGG human diseases super-pathway, and specifically “bacterial secretion system” and two-component system pathways (33, 34), indicates a community in a heightened state of activation. Additionally, mismatch repair enrichment suggests genomic stress, while streptomycin biosynthesis points to active interspecies antagonism typical of unstable ecosystems (35). These microbiome-derived functional predictions aligned with metabolomic enrichments in the sulfur amino acid pathway, such as taurine and hypotaurine metabolism and cysteine and methionine metabolism, which are hallmarks of oxidative and inflammatory stress (36). Together, these patterns reflect a gut environment lacking ecological stabilization, potentially contributing to host vulnerability through both microbial activity and metabolic output.

### Limitations

This study has several limitations. First, the small cohort size and variability in gestational age limit statistical power and increase the susceptibility to residual confounding from clinical factors such as antibiotic exposure and nutrition. Although key perinatal variables were included in baseline tables and showed no group differences, the observational design precludes causal inference. Second, all analyses were based on fecal samples; airway sampling is ethically infeasible in neonates. Thus, our findings reflect gut microbial-metabolic patterns associated with BPD risk rather than direct evidence of lung colonization. Prior work (17, 18) supports gut systemic communication, but multicompartment studies will be required to establish mechanistic links. Third, the absence of term infants limits the ability to distinguish BPD-specific signatures from prematurity-associated dysbiosis. Existing literature indicates that preterm infants naturally display reduced diversity and low Bacteroidota (26, 28), suggesting that the BPD-associated imbalance observed here may represent an amplification of prematurity-related dysbiosis. Lastly, because only three NBPD infants were resampled at day 28, longitudinal inferences, such as the observed decline in Bacteroidota, should be interpreted as preliminary. Future studies with larger, independent cohorts and integrated analysis of gut, airway, and immune compartments will be essential to validate these findings and clarify mechanistic pathways.

### Conclusions

Our integrated multiomics analysis uncovered a novel mechanistic link between gut microbial dysbiosis and the pathogenesis of BPD in preterm infants. While initial analyses revealed composition differences, deeper correlation-based investigations identified a critical functional dynamic: a significant negative association between *Streptococcus* and *Veillonella*, representing a core axis of gut microbial maturation. The enrichment of *Streptococcus* was closely associated with a host metabolic profile indicative of elevated oxidative stress characterized by increased levels of cysteic acid and may contribute to the subsequent development of BPD. This work provides new insight into the role of the gut-lung axis in BPD pathophysiology, offering both a multidimensional biomarker for early risk stratification and a potential microbial-metabolic target for future therapeutic intervention.

## Data Availability

The raw reads of 16S MiSeq data were deposited into the NCBI Sequence Read Archive database (accession no. PRJNA1293870). The raw LC-MS data have been deposited to MetaboLights (accession no. MTBLS13364).

## SUPPLEMENTAL MATERIAL

File S1 spectrum.02740-25-s0001.xlsx ASV table.

File S2 spectrum.02740-25-s0002.xlsx LEfSe LDA scores and raw *P*-values.

File S3 spectrum.02740-25-s0003.xlsx Different metabolites.

File S4 spectrum.02740-25-s0004.xlsx Metabolites in the positive ion mode.

File S5 spectrum.02740-25-s0005.xlsx Metabolites in the negative ion mode.

File S6 spectrum.02740-25-s0006.xlsx Relative abundance at the genus level.

Supplemental figures spectrum.02740-25-s0007.docx Figures S1 to S4.

Supplemental tables spectrum.02740-25-s0008.docx Tables S1 to S3.

ASM does not own the copyrights to Supplemental Material that may be linked to, or accessed through, an article. The authors have granted ASM a non-exclusive, world-wide license to publish the Supplemental Material files. Please contact the corresponding author directly for reuse.
