# Supplementary material for: Gut microbiota and metabolomic changes across preterm stages: potential associations with bronchopulmonary dysplasia
Source: Microbiol Spectr. 2026 Feb 6;14(3):e02740-25. doi: 10.1128/spectrum.02740-25 (PMC12955448; doi:10.1128/spectrum.02740-25)

**FIG S1** Venn diagrams showing shared and unique ASVs among different groups of preterm infants. ASV overlap among early (S1, *n* =13), middle (S2, *n* = 14), and late (S3, *n* = 35) preterm groups (a). Comparison of ASVs between BPD and NBPD groups at the first sampling time point (b). ASV overlap between the two sampling time points within the BPD (BPD1/BPD2, *n* = 3) group (c). ASV overlap between the two sampling time points within the NBPD (NBPD1/NBPD2, *n* = 3) group (d).


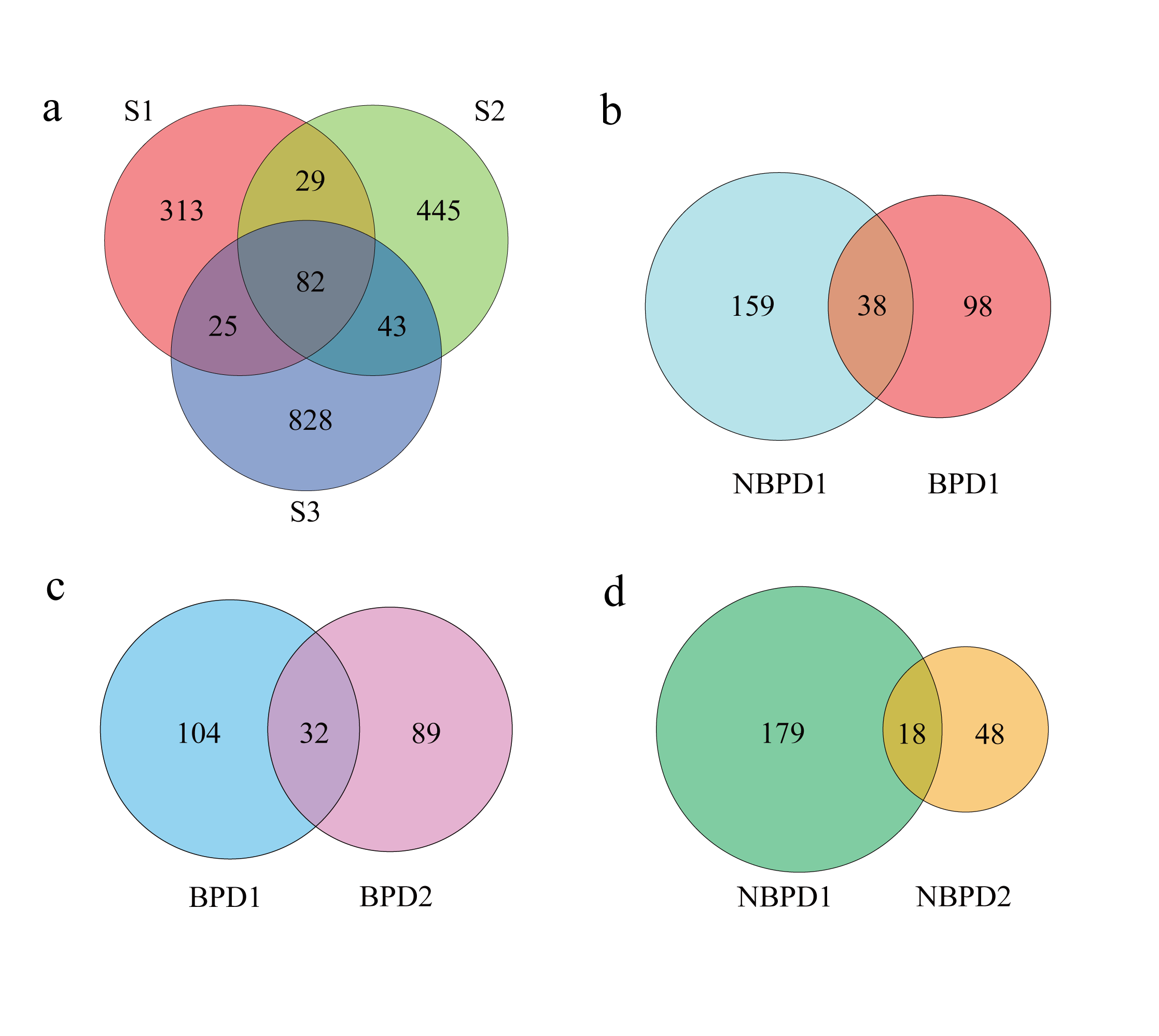


**FIG S2** Longitudinal changes in gut bacterial composition at the phylum level in BPD (a) and NBPD infants (b) (*n* =3). Individual-level trajectories of Bacteroidota abundance for BPD (c) and NBPD (d) infants, illustrating paired within-infant changes across the two sampling timepoints. Each line represents one infant. Statistical comparison was performed using the paired Wilcoxon signed-rank test. Although not statistically significant due to the small cohort size, the effect size indicates a moderate-to-large magnitude of change.


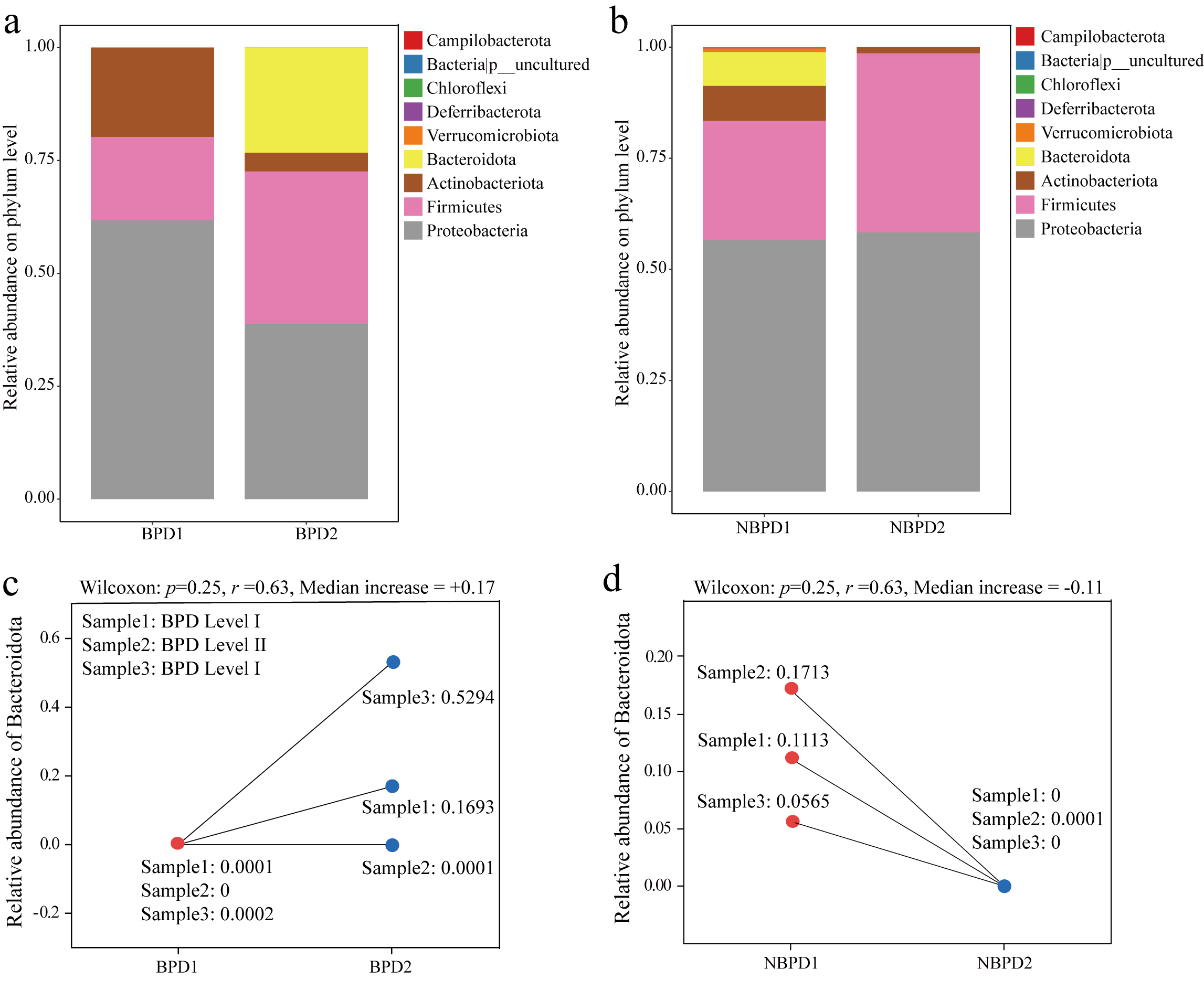


**FIG S3** Differential bacterial taxa identified by linear discriminant analysis effect size (LEfSe). (a) Taxa with significant differences in relative abundance among early (S1, *n* = 13), middle (S2, *n* = 14), and late (S3, *n* = 35) preterm infants. (b) Discriminative taxa between BPD1 and NBPD1 (*n* = 3). Only taxa with an LDA score > 2.0 and *p* < 0.05 were considered significant.


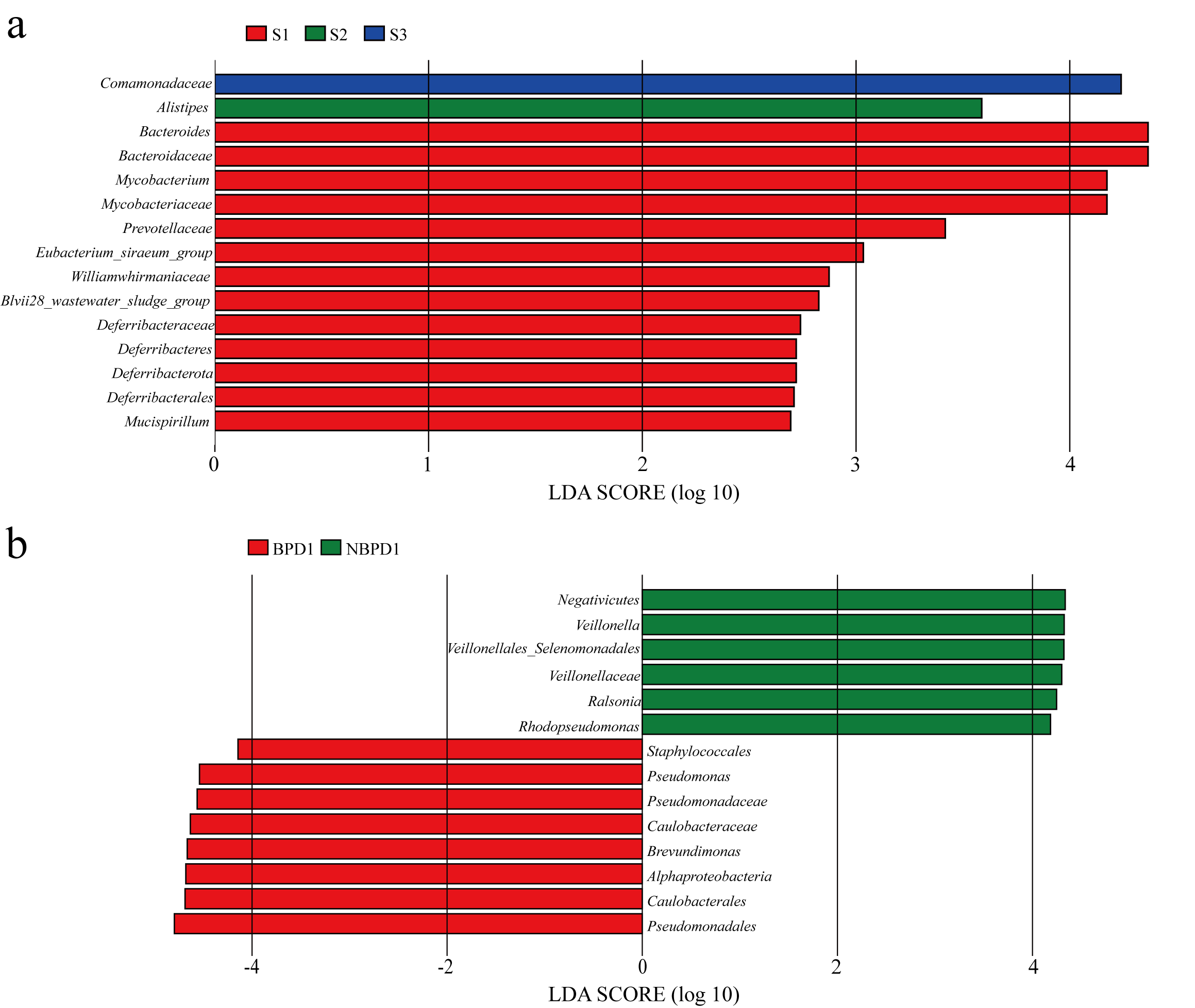


**FIG S4** Comparative analysis of gut metabolomes between preterm infants with and without BPD. (a) Principal component analysis (PCA) based on positive ion mode metabolites. (b) Volcano plot of significantly regulated metabolites under positive ion mode. (c) Bar plot of significant differential metabolites with relative abundance comparisons between BPD (BPD1+BPD2, *n* = 6) and NBPD (NBPD1+NBPD2, *n* = 6) groups. Bars represent mean ± standard deviation (SD). Statistical significance was assessed using the Wilcoxon rank-sum test, with * indicating *p* < 0.05.


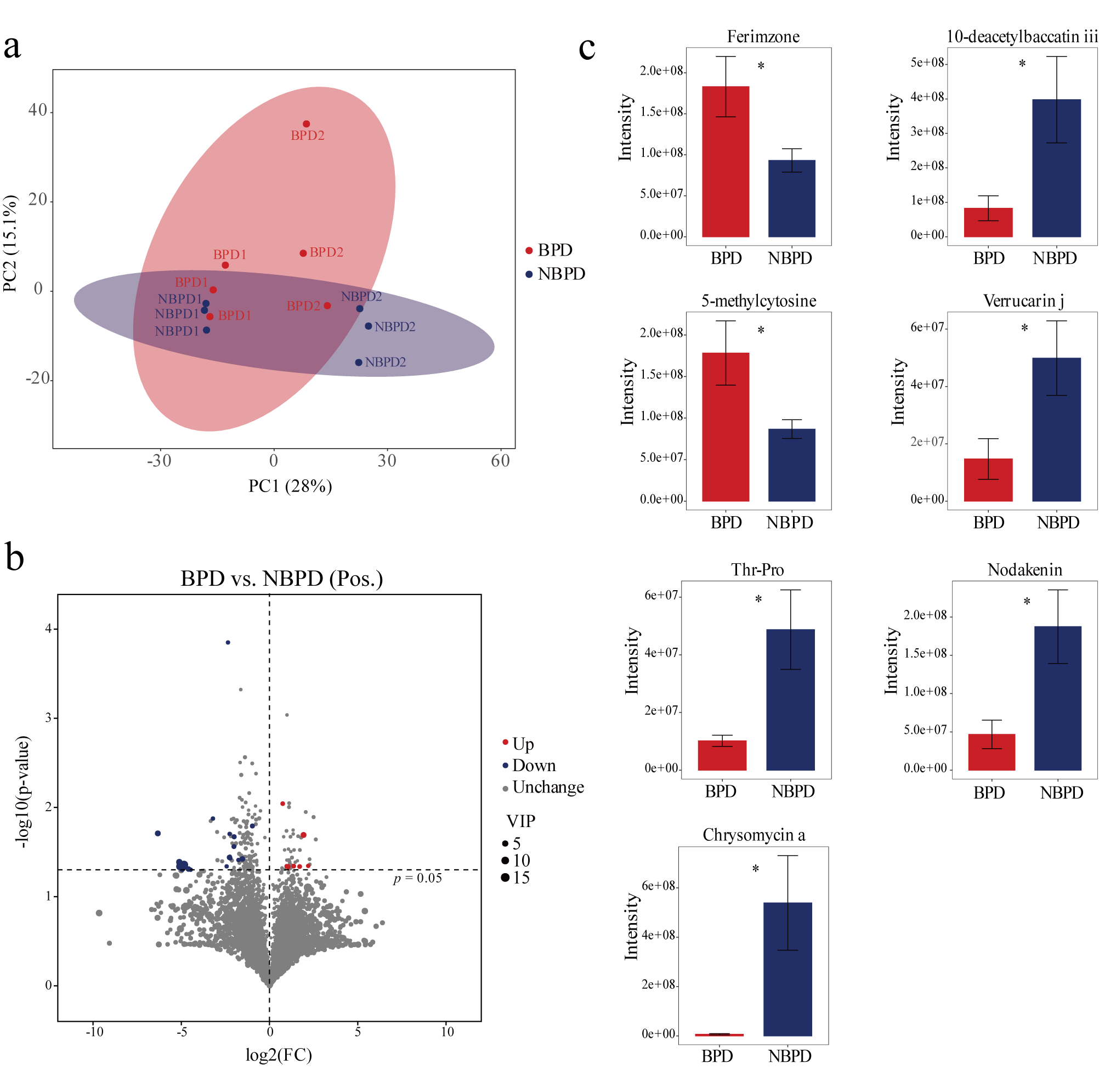

Supplement: Supplemental figures — Figures S1 to S4. [file spectrum.02740-25-s0007.docx]
