# Supplementary material for: Gut microbiota and metabolomic changes across preterm stages: potential associations with bronchopulmonary dysplasia
Source: Microbiol Spectr. 2026 Feb 6;14(3):e02740-25. doi: 10.1128/spectrum.02740-25 (PMC12955448; doi:10.1128/spectrum.02740-25)
Supplement: Supplemental tables — Tables S1 to S3. [file spectrum.02740-25-s0008.docx]

**TABLE S1** Refinements to the definition of BPD

| A premature infant (<32 weeks’ gestational age) with BPD has persistent parenchymal lung disease, radiographic confirmation of parenchymal lung disease, and at 36 weeks PMA requires 1 of the following FiO_2_ ranges/oxygen levels/O_2_ concentrations for ≥3 consecutive days to maintain arterial oxygen saturation in the 90%-95% range. | | | | | |
| --- | --- | --- | --- | --- | --- |
| Grades | Invasive IPPV* | N-CPAP, NIPPV, or nasal cannula ≥3 L/min | Nasal cannula flow of 1 ≤3 L/min | Hood O_2_ | Nasal cannula flow of <1 L/min |
| Ⅰ | - | 21 | 22-29 | 22-29 | 22-70 |
| Ⅱ | 21 | 22-29 | ≥30 | ≥30 | >70 |
| Ⅲ | >21 | ≥30 |  |  |  |
| Ⅲ(A) | Early death (between 14 days of postnatal age and 36 weeks) owing to persistent parenchymal lung disease and respiratory failure that cannot be attributable to other neonatal morbidities (eg. Necrotizing enterocolitis, intraventricular hemorrhage, redirection of care, episodes of species, etc). | | | | |

* Excluding infants ventilated for primary airway disease or central respiratory control conditions. Values are percents.

*CPAP*, continuous positive airway pressure; *IPPV*, intermittent positive pressure ventilation; *N-CPAP*, nasal continuous positive airway pressure; *NIPPV*, noninvasive positive pressure ventilation.

**TABLE S2** Neonatal birth weight, maternal delivery mode, and maternal antepartum antibiotic use

| Group | Weight (kg) | Cesarean | Eutocia | Antibiotic use | Fecal samples |
| --- | --- | --- | --- | --- | --- |
| S1 | 1.29 ± 0.25 | 5 | 5 | - | 13 |
| S2 | 1.62 ± 0.23 | 10 | 2 | Azithromycin (1); Cefazolin (1) | 14 |
| S3 | 2.17 ± 0.45 | 9 | 25 | Piperacillin (1); Cefazolin (1) | 35 |

S1: early preterm (28 weeks to 31 weeks and 6 days), S2: middle preterm (32 weeks to 33 weeks and 6 days), and late preterm (34 weeks to 36 weeks and 6 days). In S1, S2, and S3, there were 3 children, 2 children, and 1 child whose fecal samples were taken twice. The weight are presented as the mean ± standard deviation. The numbers in parentheses after each antibiotic indicate the number of mothers who received that medication prior to delivery.

**TABLE S3** Basic characteristics of mothers and infants after birth (For BPD1-BPD2; NBPD1-NBPD2)

| Group | BPD grade | Feeding method | Antibiotic use  (Children) | Antibiotic use  (Mother) | Steroid use  (Mother) |
| --- | --- | --- | --- | --- | --- |
| BPD (1) | Ⅱ | Breast milk/  Formula | Ceftazidime | Budesonide | - |
| BPD (2) | Ⅰ | Formula | Epocelin/Penicillin | Budesonide | Cefazolin |
| BPD (3) | Ⅰ | Formula | Cefotaxime | - | - |
| NBPD (1) | - | Formula | Ceftazidime | - | - |
| NBPD (2) | - | Formula | Sulperazone/Meropenem | cefuroxime |  |
| NBPD (3) | - | Formula | Meropenem/Cefoperazone-Sulbactam | Cefuroxime/Morinidazole | Budesonide |

The numbers in parentheses indicate three repetitions.
